# Comparative Analysis of Vectorial Capacity Among *Triatoma brasiliensis brasiliensis, Triatoma juazeirensis,* and Their Experimental Hybrids

**DOI:** 10.3390/microorganisms13051025

**Published:** 2025-04-29

**Authors:** Nathália Cordeiro Correia, Carlos José de Carvalho Moreira, Fernanda Oliveira Firmino, Dayse da Silva Rocha, João Paulo Sales Oliveira-Correia, Cleber Galvão, Jane Costa

**Affiliations:** 1 Laboratório de Doenças Parasitárias, Instituto Oswaldo Cruz, Fiocruz, Av. Brasil 4365, Rio de Janeiro 21040-900, Brazil; nathcor@gmail.com (N.C.C.); moreira@ioc.fiocruz.br (C.J.d.C.M.); 2 Laboratório Nacional e Internacional de Referência em Taxonomia de Triatomíneos, Instituto Oswaldo Cruz, Fiocruz, Av. Brasil 4365, Rio de Janeiro 21040-900, Brazil; fernandaoliveirafirmino@gmail.com (F.O.F.); dayseroch@gmail.com (D.d.S.R.); joao.correia@ioc.fiocruz.br (J.P.S.O.-C.); clebergalvao@gmail.com (C.G.)

**Keywords:** triatominae, hybridization zone, feeding and defecation, epidemiological importance, chagas disease

## Abstract

The existence of a natural hybridization zone of members of the *Triatoma brasiliensis* complex in Pernambuco, a Brazilian state with areas highly infested by *Trypanosoma cruzi* vectors, raised questions to be studied about the vectorial capacity of these hybrids. Recently, it was demonstrated that experimental hybrids of *T. brasiliensis brasiliensis* and *Triatoma juazeirensis* present vectorial competence superior to that of the parental species. The objective of the present study was to compare bionomic aspects related to the vectorial capacity of *T. b. brasiliensis*, *T. juazeirensis,* and their experimental hybrids. Feeding and defecation behavior patterns in fifth instar nymphs were comparatively analyzed between four groups, two parental and two hybrids, respectively: *T. b. brasiliensis*, *T. juazeirensis*, Hjb (♀ *T. juazeirensis* × ♂ *T. b. brasiliensis*), and Hbj (♀ *T. b. brasiliensis* × ♂ *T. juazeirensis*). Fifteen newly molted fifth instar nymphs from each of the mentioned groups were previously subjected to a period of fasting for 10 days and individually kept in identified bottles. In the experiment, the insects were placed in a jar containing a mouse immobilized in nylon mesh, in accordance with the guidelines of the animal ethics committee. The following variables were observed: 1-total number of feedings carried out; 2-time to start feeding; 3-duration of feeding and weight acquired; 4-defecations during feeding; 5-defecation within one minute after cessation of feeding; 6-defecation within ten minutes after cessation of feeding; 7-defecation behavior. Specimens from all groups demonstrated voracity, starting their meal immediately after contact with the mouse, and most of the insects defecated immediately after feeding, with 78% of the insects defecating within 30 s. The results obtained suggest that *T. brasiliensis*, *T. juazeirensis*, and their experimental hybrids presented bionomic characteristics compatible with the species considered good vectors in the literature. However, *T. b. brasiliensis* and *T. juazeirensis* demonstrated even more effective characteristics for *T. cruzi* transmission regarding their feeding and defecation patterns when compared to their hybrids.

## 1. Introduction

Chagas disease is a serious and neglected health problem that affects between six and seven million people worldwide, especially in Latin America [1]. As there is no vaccine or medication for the chronic phase, the most effective control is the elimination of triatomines, vectors of the etiological agent *Trypanosoma cruzi* (Chagas, 1909) [1,2,3,4]. The greatest diversity of triatomine species is found in Brazil, with 64 species recorded, of which 30 species occur in the northeastern region [5,6,7]. Most of the cases of *T. cruzi* transmission occur when humans are exposed to feces and/or urine of infected blood-sucking triatomine bugs and also by oral contamination. Therefore, understanding vector competence and capacity is crucial for effective monitoring and defining proactive control actions in endemic areas [1,8].

The *Triatoma brasiliensis* species complex comprises a group of six species and two subspecies that present distinct morphology, geographic distribution, bionomy, and epidemiological importance [9,10,11,12]. Among these, *Triatoma brasiliensis brasiliensis* Neiva, 1911, is the most relevant in epidemiological terms due to its wide distribution infesting natural and artificial ecotopes, percentages of natural infection by *T. cruzi*, and dietary eclecticism, being able to feed on human blood [9,10,11,12,13,14].

In some areas of Pernambuco, a Brazilian state, different intermediate phenotypes of this species complex were recorded [15]. Those intermediate phenotypes’ distribution coincides with the areas infested by *Triatoma brasiliensis macromelasoma* Galvão, 1956, a subspecies suggested as a hybrid product between *Triatoma b. brasiliensis* and *Triatoma juazeirensis* by Costa and Felix, 2006. Comparative morphological and molecular studies between parental species and hybrids corroborate the existence of a natural hybridization area in the state of Pernambuco and the hybrid origin of *T. b. macromelasoma* [14,15]. This subspecies was suggested as a product of homoploidal hybrid speciation, an evolutionary process for the first time described for triatomines [15,16].

Those distinct phenotypes showed a low percentage of natural infection in the feces when compared to the prevalence observed for members of the *T. brasiliensis* complex, suggesting low susceptibility to infection by *T. cruzi* [17]. Conversely, Herrera-Aguilar et al. [18] and Martínez-Hernandez et al. [19] mentioned that natural hybrids of *Triatoma dimidiata* (Latreille, 1811) would present higher infection rates than their parental species. As was observed for the experimental hybrids between *T. b. brasiliensis* and *T. juazeirensis* [20].

Previous studies on the vector competence of triatomines observed parameters such as time elapsed since the insect started feeding, and for the blood ingestion, the contact with the host and time elapsed for defecation. All these variables are important to better understand the transmission of *T. cruzi*, allowing estimation of the epidemiological importance of the distinct vector species [21,22].

Zeledón et al. [23] state that species that defecate up to 10 min after a blood meal can be considered potentially effective vectors for the transmission of *T. cruzi*, since there is a greater possibility that the insects are still in contact with the host. Even if insects do not defecate during feeding, it is important to consider post-meal behavior by checking whether the vector species releases feces near or far from the food source [24].

There are triatomine species naturally highly infected by *T. cruzi* that are not considered good vectors due to their feeding and defecating behaviors, such as *Triatoma rubrofasciata* (De Geer, 1773). This species, despite being found naturally infected and infesting artificial ecotopes, rarely defecates during a blood meal [25].

In regions where Chagas disease is not endemic, few species of triatomines are able to invade homes, and the main characteristic of these insects is that they do not defecate immediately after feeding, demonstrating low vector potential [26]. This is the case of *Triatoma vitticeps* (Stal, 1859), which frequently invades homes and generally presents high rates of *T. cruzi* infection [27]. However, its bionomical characteristics indicate low vector competence [21,28], which may explain the low incidence of Chagas disease in regions where *T. vitticeps* occurs [29].

Another relevant characteristic is the persistence of triatomines in obtaining a blood meal, as some species quickly encounter the host and do not leave until they are completely engorged. Host accessibility is a key factor influencing the blood-foraging behavior of triatomines. This characteristic can be studied under laboratory conditions, shedding light on the insect behavior when in contact with the host, helping to understand and estimate the vectorial potential of a triatomine species [30,31,32].

Aiming to understand the bionomic characteristics of *T. cruzi* vectors, such as *T. b. brasiliensis*, *T. juazeirensis,* and their experimental hybrids, this research records, analyzes, and comparatively discusses their feeding and defecation behaviors under laboratory conditions.

## 2. Materials and Methods

Samples. The specimens of *T. b. brasiliensis* (Caicó—Rio Grande do Norte, 06° 27′ S 37° 05′ W) and *T. juazeirensis* (Curaça—Bahia, 09°12′ S 39°83′ W) were collected and maintained under controlled temperature conditions (52–70% relative humidity and 23–24.8 °C) at the Laboratório Interdisciplinar em Vigilância Entomológica de Diptera e Hemiptera of the Instituto Oswaldo Cruz. Insects were identified according to taxonomic keys proposed by Lent and Wygodzinsky [33] and Costa et al. [34].

Fourth instar nymphs were randomly separated from each of the colonies and fed weekly on Swiss Webster mice in accordance with the guidelines of the animal ethics committee (CEUA license LW 18-11). After ecdysis from the fourth to the fifth instar, the nymphs were separated as follows: one group was identified by sex and separated to form five couples and obtain virgin hybrid adults, and the second was kept fasting for 10 days with the aim of studying behavioral patterns of feeding and defecation. In the eventual death of some insect, another one replaced the dead one.

The insects were kept in identified plastic containers (14 × 14 × 15 cm) with the bottom lined with filter paper and wide strips of paper in an accordion shape, increasing the inner surface and allowing the absorption of moisture caused by insect waste (feces and urine).

The pairs consisted of females of *T. b. brasiliensis* and males of *T. juazeirensis* and the reverse combination. The insects were fed weekly with Swiss Webster mice. The F1 hybrid specimens obtained were monitored until fifth instar nymphs. A total of 30 hybrid specimens from each cross were selected. The experiment followed the methodology of Correia et al. [20].

**Figure 1:**
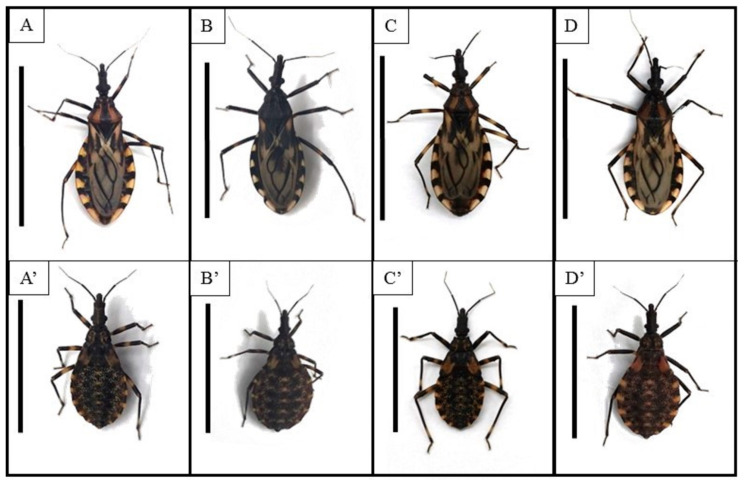
General aspect in dorsal view of specimens of *Triatoma b. brasiliensis* ((**A**), adult and (**A’**), fifth instar nymph), *Triatoma juazeirensis* (**B**,**B’**), Hbj [hybrid of *T. b. brasiliensis* ♀ × *T. juazeirensis* ♂] (**C**,**C’**), and Hjb [*T. juazeirensis* ♀ × *T. b. brasiliensis* ♂] (**D**,**D’**). Images from Correia et al. [20].

Feeding and defecation behavior. Fifteen newly molted fifth instar nymphs of *T. b. brasiliensis*, *T. juazeirensis*, and their reciprocal hybrids were fasted for 10 days (Figure 1). The insects were separated into individually labeled Borrel vials lined with filter paper. Subsequently, the nymphs were fed. The parameters analyzed were the time elapsed for starting and ending the meal and the timing and behavior of defecation. These observations were performed weekly until the imaginal molt.

The specimens were placed close to the food source, and the start of feeding was timed from the moment the proboscis was inserted into the food source. The duration of feeding was calculated, discounting any interruptions. Specimens that did not feed were observed for 15 min. Insects that fed continued to be observed for 10 min to quantify the completion of defecation. Specimens were weighed on a precision scale (Libor AEG, Shimadzu, Kyoto, Japan) before and after feeding to estimate blood ingestion.

Statistical Analysis. The analyses of behavioral parameters (feeding and defecation) were evaluated using the nonparametric Kruskal–Wallis test (SPSS version 22), ANOVA, and paired *t*-test to compare groups. Kendall’s correlation test was applied using nonparametric data such as mean feeding time, defecation time, and weight over five weeks (Supplementary Tables S1 and S2). The analyses were performed using RStudio v4.4.2 with the “dplyr” and “lubridate” packages.

## 3. Results

The analysis of the fifth instar nymphs feeding showed that during the first two weeks, the insects avidly sought the food source. From the third to the fifth week, this demand decreased, except for Hjb and *T. b. brasiliensis*, which remained constant (Table 1).

All specimens showed voracity in the search for a food source and an immediate start to blood supply. In the first week, *T. juazeirensis* showed an average time of 55 s to start feeding, Hbj 1 minute and 24 s, and Hjb and *T. b. brasiliensis* 1 minute and 52 s and 1 minute and 53 s, respectively (Supplementary Tables S1 and S2).

**Figure 2:**
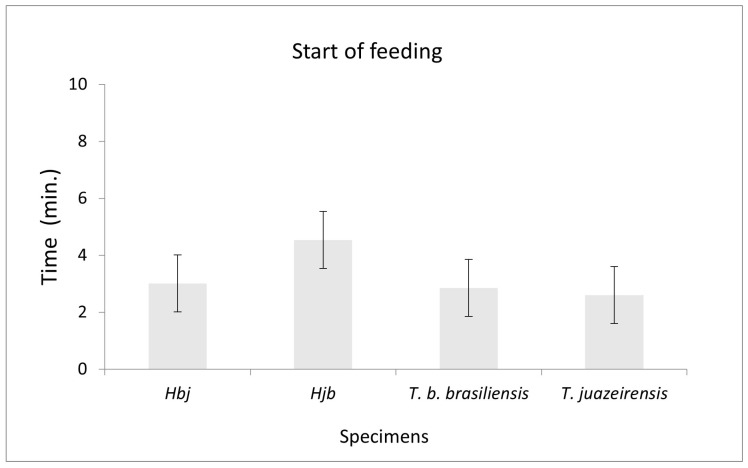
Average time (min) of the beginning of feeding of the experimental hybrids Hbj (hybrid of *T. b. brasiliensis* ♀ × *T. juazeirensis* ♂), Hjb (*T. juazeirensis* ♀ × *T. b. brasiliensis* ♂), and the parental specimens *Triatoma b. brasiliensis* and *Triatoma juazeirensis* during five weeks. Feeding start times among the species were significantly different according to ANOVA F (3, 36) = 2.91, *p* = 0.0479). A paired *t*-test revealed a significant difference between Hjb and *T. b. brasiliensis* (t = 2.57, df = 18, *p* = 0.019).

Figure 2 shows the average time over five weeks. From the second week onwards, specimens of *T. juazeirensis* and *T. b. brasiliensis* showed a behavior of approaching the food source and starting to feed in less time compared to the hybrid ones. Over the weeks, it was observed that the insects took longer to start feeding.

The third week of feeding showed significant differences (Kruskall–Wallis, x2 = 71.533, *p* = 0.013), according to the ANOVA test (Tukey and Student–Newman–Keuls post hoc test). There was a delay in starting feeding compared to other weeks. Meanwhile, it was observed that hybrids took longer to make contact with the food source compared to *T. b. brasiliensis* and *T. juazeirensis*.

The time during feeding and the amount of blood ingested are shown in Table 2. *Triatoma juazeirensis* presented an average of 36 min (minimum = 2 min; maximum = 121 min), Hbj with 37 min (minimum = 3 min; maximum = 169 min), *T. b. brasiliensis* with 41 min (minimum = 46 s; maximum = 156 min), and Hjb with 48 min (minimum = 2 min; maximum = 184 min). Based on these data, statistical analysis showed no significant differences for the feeding duration parameter between groups.

**Figure 3:**
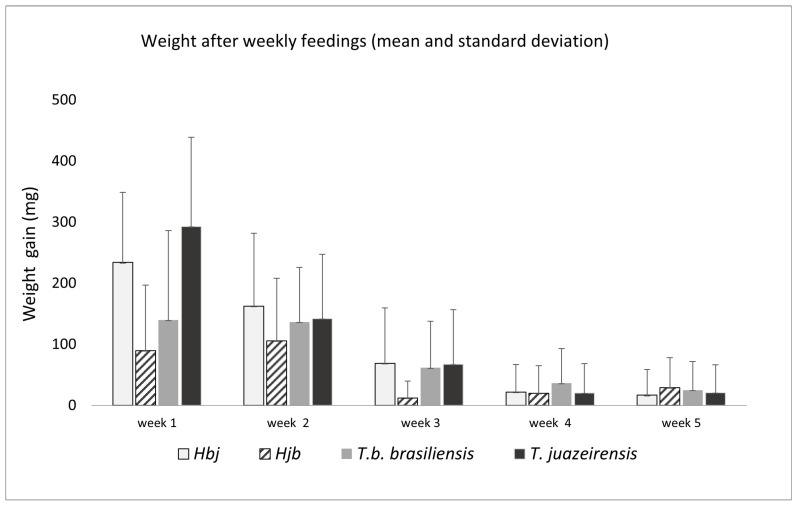
Mean and standard deviation of weight gain (mg) after feeding observed from the first to the fifth week of *T. b. brasiliensis* and *T. juazeirensis* and their experimental hybrids (Hbj [hybrid of *T. b. brasiliensis* ♀ × *T. juazeirensis* ♂] and Hjb [*T. juazeirensis* ♀ × *T. b. brasiliensis* ♂]). Paired *t*-tests showed a greater weight gain in Hbj hybrids compared to the groups during week 1: Hjb (t = 72.35, df = 29, *p* = 0.0016), T. b. brasiliensis (t = 55.18, df = 25, *p* = 0.0412), and T. juazeirensis (t = 73.30, df = 18, *p* = 0.0096).

The average weight gained in the first five weeks was as follows: *T. b. brasiliensis* (80 mg), *T. juazeirensis* (109 mg), Hjb (51 mg), and Hbj (101 mg) (Figure 3). The first week was the period with the highest blood consumption, with a significant difference of X^2^ = 180,521.49, *p* < 0.01. However, through the post hoc test, we observed that *T. b. brasiliensis* and Hjb obtained lower intake means compared to *T. juazeirensis* and Hbj. Over time, the insects no longer sought the food source with the same frequency, and most specimens that started feeding remained for a short time, resulting in a reduction in weight gain (Figure 3).

**Figure 4:**
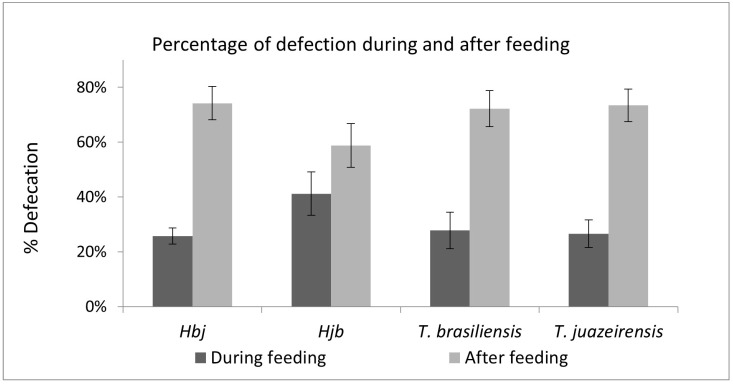
Percentage of defecations that occurred during the meal (dark gray bar) and after the meal (light gray bar). In hybrids: Hbj (hybrid of *T. b. brasiliensis* ♀ × *T. juazeirensis* ♂ [n = 30]); Hjb (*T. juazeirensis* ♀ × *T. b. brasiliensis* ♂ [n = 12]); and in parental specimens: *T. b. brasiliensis* (n = 30) and *T. juazeirensis* (n = 30). n= number of specimens.

Hjb specimens performed more defecations during feeding compared to the Hbj and parental species (Figure 4). Among the others, *T. b. brasilienis*, *T. juazeirensis*, and Hbj were similar.

Some specimens began feeding immediately after contact with the blood source, the longest time being 6 min 31 s. At first, about 78% of the insects defecated within 30 s. In the first week, all individuals defecated during feeding, with only five insects defecating within 30 s.

*Triatoma juazeirensis* had the shortest time interval between feeding and defecation, with the minimum time being 1 s and the maximum time being 3 min 14 s, with 82% of the specimens defecating within a period of 30 s. No specimen defecated during feeding in the third week of observation.

Hbj defecated during feeding faster compared to *T. b. brasiliensis* and *T. juazeirensis*, defecating in a minimum period of <1 s and a maximum of 6 min 31 s, with 81% of the specimens defecating within 30 s of the feeding interval. Of the Hjb group, 60% of the specimens defecated within 30 s. The minimum defecation period during feeding presented by this group was also <1 s and a maximum of 3 min 26 s.

**Figure 5:**
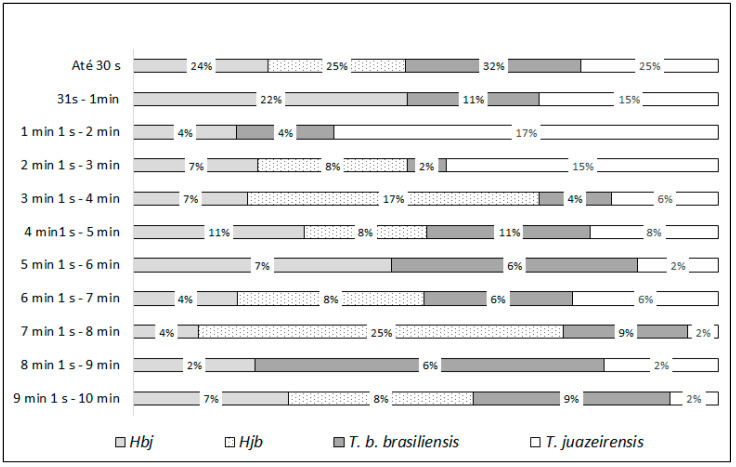
Percentage of defecations of specimens observed up to 10 min after eating, of *T. brasiliensis* species complex parental specimens (*T. b. brasiliensis* and *T. juazeirensis*) and their experimental hybrids (Hbj [hybrid of *T. b. brasiliensis* ♀ × *T. juazeirensis* ♂] and Hjb [*T. juazeirensis* ♀ × *T. b. brasiliensis* ♂]).

After feeding, the parental and hybrid specimens defecated in less than one minute. The percentage of specimens that defecated within 10 min after feeding was also observed (Figure 5). Over time, the amount of food ingested and defecations decreased, with the interval between them increasing. We can observe that from the fourth week of feeding, defecations begin to occur after 3 min (Figure 5).

It is worth noting that in the second week of feeding, most of the specimens studied eliminated more liquid. After a period of fasting, when they fed again, they generally eliminated feces or liquid mixed with feces.

The specimens presented the following behaviors during defecation in relation to the mouse: (a) close and not moving away to defecate; (b) close, defecating and dragging the abdomen along the bottom of the container, leaving a trail of feces; (c) moving away; and (d) close and rotating the abdomen approximately 50 to 360°. The most common behaviors observed in Hjb and Hbj were the following: (a) turning approximately 180° and defecating and (b) moving backwards, turning approximately 180°, and defecating. The species *T. b. brasiliensis* and *T. juazeirensis* presented similar defecation behavior, being closer to the mouse.

The correlation analysis between feeding and defecation times showed longer feeding and shorter defecation times for HBJ. For the variables feeding time and weight, it was also shown that the hybrids were able to gain weight in shorter feeding times. Thus, with such correlations, we observed that the hybrids were able to ingest more blood in shorter feeding times. On the other hand, the correlation between defecation time and weight highlighted the HBJ specimens as defecating faster when they were heavier. All correlation values between the parentals and hybrids are shown in Table 3.

## 4. Discussion

Studies on the vector competence of triatomines are relevant, allowing the characterization of the good vectors of *T. cruzi*. Through the observation of several parameters, such as time elapsed since the insect started feeding, for the blood ingestion, for the contact with the host, and time elapsed for defecation, it is possible to better understand and estimate the epidemiological importance of the distinct vector species [21,22,23,24,25].

Our data corroborated previous studies on feeding and defecatory behavior, demonstrating significant differences among Triatominae species [23,35,36,37,38]. It was observed during the first week of feeding, after a 10-day fasting period, that *T. juazeirensis* nymphs presented the shortest time ($\bar{x}$ = 55 s) to start feeding and *T. b. brasiliensis* nymphs the longest one ($\bar{x}$ = 1 min 53 s). The voracity an insect displays in its search for food can be measured by recording the time elapsed between the supply of the food source and the insect bite [23,35,36]. According to Araújo et al. [38], fifth instar nymphs require more intense nutritional activity, becoming more avid for food and ingesting larger quantities of blood. Almeida et al. [37] observed that the time elapsed for fifth instar nymphs of *Triatoma rubrovaria* (Blanchard, 1846) to begin feeding was shorter compared to other instars of the species.

Almeida et al. [37] found that *T. rubrovaria* fifth instar nymphs fasted for 20 days and had an average time of 28 s to start feeding. Guarneri et al. [39] demonstrated that *T. brasiliensis*, *Triatoma infestans* (Klug 1834), and *Triatoma pseudomaculata* Corrêa e Espínola, 1964 nymphs fasted for 30 days and started feeding within 20 s. Compared to our results, the average time elapsed for the specimens to start feeding was longer, but the time the specimens were kept fasting was shorter. However, the minimum time elapsed for insects to start feeding was the same in all groups studied, one second or immediately, and coincides with the other studies mentioned above [37,39].

The approach of the insect to the food source and the insertion of its mouthparts into the skin of the mouse is the moment that may cause greater perception by the host. Therefore, insects that take longer to probe the blood vessel may have greater difficulty in obtaining blood from the host [40]. Our results indicate that this did not occur for any of the groups studied, as all of them began feeding after an average of three minutes.

Lima-Neiva [41] observed that the fifth instar nymphs of *Triatoma sherlocki* Papa et al. 2002, another species of the *T. brasiliensis* complex, took around nine minutes to start the blood meal, an average much longer than those studied in the present work. This can be explained by the fact that the specimens had not attended any period of fasting and had nutritional reserves from the last meal in their stomach [42].

*Triatoma b. brasiliensis* is highly voracious in its natural environment, always searching for food, and often defecating while still in contact with the food source, demonstrating that it is a competent vector [43]. Under laboratory conditions, this species can feed for long periods without interruption and often rapidly defecates or even while feeding [39,43]. We observed that the specimens took the shortest time to approach the food source and start feeding, showing no difference from those recorded for *T. juazeirensis*. It is worth highlighting that the duration of feeding can take from a few minutes to more than 30 min. It depends on several factors: type of host, insect species, evolutionary stage, and volume of blood ingested [38].

The duration of feeding time also depends on the developmental stage of the nymphs. The fifth instar nymphs are the ones that remain feeding for the longest time, due to their high blood ingestion capacity. Ecdysis can be achieved after just one feeding and usually occurs between 6 and 30 days. Reports indicate that some species require more than one feeding to promote ecdysis [23,41,44]. During the first weeks of observation, some specimens remained feeding for hours, such as *T. juazeirensis* (2 h 01 min), *T. b. brasiliensis* (2 h 36 min), Hbj (2 h 49 min), and Hjb (3 h 4 min). Specimens of *T. juazeirensis* were the first to undergo imaginal molt, with 60%, followed by *T. b. brasiliensis* (27%), Hbj (23%), and Hjb (23%). However, the results indicate *T. juazeirensis* required less feeding time to perform ecdysis.

Most of the specimens studied fasted for more than two weeks. Feeding is essential for the maintenance of the parasite, since the parasite density rate decreases when the triatomines go through long fasting periods. According to Kollien and Schaub [45], after three months of fasting, approximately 90% of the parasites die in the insect’s digestive tract. According to Guarneri et al. [39], analyzing the engorgement rate and population density of vectors in human dwellings showed *T. infestans* fed faster, followed by *T. b. brasiliensis*. The feeding pattern of *T. infestans* may directly influence the dynamics of waste elimination and, consequently, the transmission of *T. cruzi*, since insects that ingest larger quantities of blood tend to defecate in less time [40]. However, this correlation was not observed in the present study, since *T. b. brasiliensis*, which presents higher domiciliary rates than *T. juazeirensis*, was recorded with a lower average blood ingestion and longer feeding duration than the latter. This observed difference may occur because species kept in laboratory conditions may present distinct behaviors compared to insects in nature [10,28,46].

Triatomines can ingest large amounts of blood, which can increase their original weight by 6 to 12 times. The amount of blood ingested can directly influence their development, infection rates, and the fecundity of females [42,47]. In contrast, species highly efficient in their diuretic process might rapidly eliminate the infective forms of *T. cruzi*. For example, *Rhodnius* nymphs can lose six times their body mass in six hours [48]. According to Galvão et al. [44], species that defecate during the blood meal or immediately after the meal ends would be more effective in transmitting the parasite. Rocha et al. [49] demonstrated, through laboratory observations of *Rhodnius pictipes Stål 1872*, that most defecations directly occurred on the host, very close to the biting site. These differences explain the effectiveness shown by some species and demonstrate the vectorial capacity of the species in transmitting the Chagas disease etiological agent [36].

Almeida et al. [37] found that 60% of *T. rubrovaria* fifth instar nymphs defecated within 2 min and 30 s, standing out from the other instars. In addition to that, *T. rubrovaria* specimens defecated during feeding and in less than seven minutes, being faster than the species and hybrids analyzed in the present study.

Among the species studied by Dias [35], *T. infestans* specimens defecated during feeding in less than 10 min, suggesting effective behavior for transmitting *T. cruzi*. Although in this experiment not all specimens defecated in less than 10 min during feeding, the majority defecated within 30 s, being 82% for *T. juazeirensis*, 81% for Hbj, 78% for *T. b. brasiliensis*, and 60% for Hjb. Zeledón et al. [23] observed that *R. prolixus* nymphs defecated faster than *T. infestans* and *Triatoma dimidiata* (Latreille, 1811), in just 1 min (70–90%). In the present study, *T. b. brasiliensis* was the species that defecated the most until one minute.

In addition to that, it was observed that many insects defecated more than once during the same feeding, especially in the first week. One specimen of *T. juazeirensis* interrupted the feeding to defecate four times, with a minimum time of 1 s. Almeida et al. [37] observed that females of *T. rubrovaria* proved to be the most efficient at defecating. The authors observed a female that defecated three times during feeding and once after feeding within 40 s.

Triatomines eliminate urine and feces separately or mixed, depending on the insect’s diet [50]. All triatomine waste, both urine and feces, might contain the infective form of the parasite. Urine not only presents a predominance of trypomastigote forms, but it is also practically free of contamination by fungi and bacteria [51]. From the second week onwards, hybrids and parental species eliminated more urine and urine together with feces. After a period of fasting, when they turn to eating, they usually eliminate feces or urine with feces. Crocco and Catalá [52] observed that during the first 10 min after feeding, *T. sordida* females eliminate urine more frequently and quickly than males and nymphs. This behavior is indicative of a profuse diuresis process that occurs after blood feeding [53].

Heitzmann-Fontenelle [54] found that *T. pseudomaculata* only defecated on the host when it reached fullness, rotating the body 80°, eliminating feces close to the biting site, increasing the chance of *T. cruzi* transmission. To perform defecation, *T. sherlocki*, a member of the *T. brasiliensis* complex, generally rotates its body (turns of 90°, 160°, 170°, and 180°), positioning the apex of the abdomen close to or in the region of the bite, and then defecates. The fifth instar was the one with the highest percentage of specimens presenting this behavior [41]. In the present study, the hybrids and parental species also showed the behavior of turning approximately 180° and defecating close to the biting site, dragging their abdomen along the bottom of the pot.

All groups studied showed the behavior of defecating while still close to the food source, and many of them were observed defecating on top of the mouse or moving from 0.5 cm to a maximum of 7 cm away from the food source. The majority who defecated 0.5 to 3 cm away were *T. b. brasiliensis* and *T. juazeirensis*. Almeida et al. [37] also found that the majority of *T. rubrovaria* specimens (69%) defecated at less than three cm from the bite site and in less than one minute. In the present study, as it was possible to monitor the feeding and defecation behaviors of some specimens for a longer period, it was verified that *T. juazeirensis* returned to defecating after the sixth week, when the other groups studied only fed without defecating. It was also seen that this species could molt faster than the other groups studied. Guarneri et al. [39] observed that some species seek more avidly to nourish themselves, perhaps because of their faster metabolism.

The fact that hybrid insects take longer to defecate than their parental species may compromise the chances of these specimens successfully transmitting the parasite to a susceptible host, although there were reports that some of the specimens were able to defecate within a few seconds.

Experimental assessments are always relative, requiring a combined analysis of several factors studied, such as the insect’s susceptibility to the parasite; their eating behavior allowing the rapid elimination of feces; rate of blood ingestion and defecation with contaminated excreta still in direct contact with the host’s skin; and number of blood meals performed [44]. In addition to these aspects, there are other relevant factors, such as household density, affinity with the host, and the degree of adaptation to the human dwellings [46,50].

## 5. Conclusions

Our study comparing *T. b. brasiliensis*, *T. juazeirensis,* and their reciprocal hybrids was carried out weekly from the fifth instar until the imaginal molt. Insects from all groups demonstrated voracity, starting their meal immediately after contact with the mouse. It was observed that the great majority of insects defecated immediately after the start of feeding and that 78% of the insects defecated within 30 s. The results obtained suggest that both parental species and hybrids presented bionomic characteristics compatible with other species considered as good vectors in the literature. However, the parental species, regarding their feeding and defecation behaviors, proved to be even more effective for propitiating the transmission of *T. cruzi.*

## Figures and Tables

**Table 1:** Percentage of weekly meals of *T. b. brasiliensis* and *T. juazeirensis* and their experimental hybrids (Hbj [hybrid of *T. b. brasiliensis* ♀ × *T. juazeirensis* ♂] and Hjb [*T. juazeirensis* ♀ × *T. b. brasiliensis* ♂]).

Species	N	Weeks				
		1st	2nd	3rd	4th	5th
Hbj	30	100%	87%	63%	33%	27%
Hjb	12	83%	100%	50%	50%	50%
*T. b. brasiliensis*	30	77%	93%	77%	57%	57%
*T. juazeirensis*	30	97%	93%	47%	37%	30%

N = number of specimens.

**Table 2:** Number of specimens (n), average time ($\bar{x}$), standard deviation (SD), and amplitudes: minimum (Min) and maximum (Max), in minutes, of the duration of feeding carried out during five weeks of the number of specimens (n) of *Triatoma b. brasiliensis*, *Triatoma juazeirensis,* and their hybrids (Hbj [hybrid of *T. b. brasiliensis* ♀ × *T. juazeirensis* ♂] and Hjb [*T. juazeirensis* ♀ × *T. b. brasiliensis* ♂]).

Species																				
*Triatoma b. brasiliensis*						*Triatoma juazeirensis*					Hbj					Hjb				
				Amplitude					Amplitude					Amplitude					Amplitude	
Weeks	N	$\bar{x}$	SD	Min	Max	n	$\bar{x}$	SD	Min	Max	n	$\bar{x}$	SD	Min	Max	n	$\bar{x}$	SD	Min	Max
1	30	48.5	36.1	8.1	156.4	30	56.2	34.9	12.6	121	30	43.1	31.6	9.4	169.2	12	65.7	47.2	22.8	184.3
2	30	42.7	32.7	2.8	150.6	30	28.7	15	0.3	64.4	30	43.1	25	11	129.9	12	47.2	42.1	11.1	166.9
3	30	43.4	47.1	3.1	213.8	30	35.6	16.2	14.5	72.2	30	31	25.6	6.8	99.4	12	66.4	64.3	2.5	154.1
4	30	41.2	42.7	3.8	140.6	30	12.1	7.4	5	29.7	30	26.6	21.2	4.5	64.6	12	30.6	18.2	10	58.5
5	30	27.4	26.4	0.3	81.3	30	21.3	17.4	0.3	46.5	30	20.3	17.2	2.9	50.4	12	28.4	17.2	10.9	58.5

**Table 3:** Correlation between the variables feeding time, defecation, and weight of *T. b. brasiliensis* and *T. juazeirensis* and their experimental hybrids (Hbj [hybrid of *T. b. brasiliensis* ♀ × *T. juazeirensis* ♂] and Hjb [*T. juazeirensis* ♀ × *T. b. brasiliensis* ♂]).

	Ft × Td		Ft × W		Td × W	
	Correlation	*p*-Value	Correlation	*p*-Value	Correlation	*p*-Value
*T. b. brasiliensis*	−0.803	0.085	−0.671	0.117	0.894	0.036
*T. juazeirensis*	−0.600	0.236	−0.738	0.077	0.738	0.077
HBJ	−0.949	0.020 *	−0.939	0.023 *	1	0.019 **
HJB	−0.130	0.805	−0.920	0.025 *	−0.105	0.801

*p*-values: ** *p* < 0.01, * *p* < 0.05, *p* < 0.1; Ft: feeding time; Td: defecation time; W: weight.

## Data Availability

The original contributions presented in this study are included in the article/Supplementary Material. Further inquiries can be directed to the corresponding author.

## Supplementary Materials

The following supporting information can be downloaded at: https://www.mdpi.com/article/10.3390/microorganisms13051025/s1. Table S1: Relationship of averages calculated over the weeks for food, defecation and weight; Table S2: Dataset on feeding time and defecation quantity of specimens.
